# Transition in the etiology of liver cirrhosis in Japan: a nationwide survey

**DOI:** 10.1007/s00535-019-01645-y

**Published:** 2019-11-25

**Authors:** Hirayuki Enomoto, Yoshiyuki Ueno, Yoichi Hiasa, Hiroki Nishikawa, Shuhei Hige, Yasuhiro Takikawa, Makiko Taniai, Toru Ishikawa, Kohichiroh Yasui, Akinobu Takaki, Koichi Takaguchi, Akio Ido, Masayuki Kurosaki, Tatsuya Kanto, Shuhei Nishiguchi, Japan Etiology of Liver Cirrhosis Study Group in the 54th Annual Meeting of JSH

**Affiliations:** 1 Division of Hepatobiliary and Pancreatic Disease, Department of Internal Medicine, Hyogo College of Medicine, Mukogawa-cho 1-1, Nishinomiya, Hyogo 663-8501 Japan; 2 Department of Gastroenterology, Yamagata University Faculty of Medicine, Yamagata, Japan; 3 Department of Gastroenterology and Metabology, Ehime University Graduate School of Medicine, Toon, Japan; 4 Center for Clinical Research and Education, Hyogo College of Medicine, Nishinomiya, Japan; 5 Department of Hepatology, Sapporo Kosei General Hospital, Sapporo, Japan; 6 Division of Hepatology, Department of Internal Medicine, Iwate Medical University, Morioka, Japan; 7 Internal Medicine, Institute of Gastroenterology, Tokyo Women’s Medical University, Tokyo, Japan; 8 Department of Gastroenterology and Hepatology, Saiseikai Niigata Daini Hospital, Niigata, Japan; 9 Department of Gastroenterology and Hepatology, Kyoto Prefectural University of Medicine, Kyoto, Japan; 10 Department of Gastroenterology and Hepatology, Dentistry and Pharmaceutical Sciences, Okayama University Graduate School of Medicine, Okayama, Japan; 11 Department of Hepatology, Kagawa Prefectural Central Hospital, Takamatsu, Japan; 12 Digestive and Lifestyle Diseases, Kagoshima University Graduate School of Medical and Dental Sciences, Kagoshima, Japan; 13 Department of Gastroenterology and Hepatology, Musashino Red Cross Hospital, Musashino, Japan; 14 Hepatitis Information Center, The Research Center for Hepatitis and Immunology, National Center for Global Health and Medicine, Tokyo, Japan

**Keywords:** Viral hepatitis, Cirrhosis, Etiology, Nationwide survey

## Abstract

**Background:**

To assess the recent real-world changes in the etiologies of liver cirrhosis (LC) in Japan, we conducted a nationwide survey in the annual meeting of the Japan Society of Hepatology (JSH).

**Methods:**

We investigated the etiologies of LC patients accumulated from 68 participants in 79 institutions (*N* = 48,621). We next assessed changing trends in the etiologies of LC by analyzing cases in which the year of diagnosis was available (*N* = 45,834). We further evaluated the transition in the real number of newly identified LC patients by assessing data from 36 hospitals with complete datasets for 2008–2016 (*N* = 18,358).

**Results:**

In the overall data, HCV infection (48.2%) was the leading cause of LC in Japan, and HBV infection (11.5%) was the third-most common cause. Regarding the transition in the etiologies of LC, the contribution of viral hepatitis-related LC dropped from 73.4 to 49.7%. Among the non-viral etiologies, alcoholic-related disease (ALD) and nonalcoholic steatohepatitis (NASH)-related LC showed a notable increase (from 13.7 to 24.9% and from 2.0 to 9.1%, respectively). Regarding the real numbers of newly diagnosed patients from 2008 to 2016, the numbers of patients with viral hepatitis-related LC decreased, while the numbers of patients with non-viral LC increased.

**Conclusions:**

HCV has remained the main cause of LC in Japan; however, the contribution of viral hepatitis as an etiology of LC is suggested to have been decreasing. In addition, non-viral LC, such as ALD-related LC and NASH-related LC, is suggested to have increased as etiologies of LC in Japan.

**Electronic supplementary material:**

The online version of this article (10.1007/s00535-019-01645-y) contains supplementary material, which is available to authorized users.

## Introduction

Liver cirrhosis (LC) due to chronic liver diseases is a health concern worldwide. LC has various etiologies, including hepatitis B virus (HBV) infection, hepatitis C virus (HCV) infection, alcohol intake, nonalcoholic fatty liver disease, and autoimmune liver diseases [1]. In many Asian and African countries, HBV infection is the most common cause of LC, and HCV is globally responsible for the development of LC in other regions. Among the liver diseases, viral hepatitis is a serious global health problem that causes 1.46 million deaths per year [2].

Because of the clinical relevance of viral hepatitis, the World Health Assembly released the Global Health Sector Strategy (GHSS) on viral hepatitis 2016–2021. The GHSS aims to reduce the incidence of hepatitis by 90% and hepatitis-associated mortality by 65% and to eliminate viral hepatitis as a major public health threat by 2030 [3, 4]. Because of the high prevalence of viral hepatitis, the Ministry of Health, Labour and Welfare (MHLW) legislated a national program, and various measures for the prevention and treatment of viral hepatitis have been intensively conducted since 2008, around eight years before the commencement of the GHSS [5]. As a national project, approximately 20 billion yen/180 million US dollars per fiscal year have been allocated to the hepatitis virus-related measures.

In light of the continuous national measures and recent advances in antiviral therapies [6–8], the rate of LC due to viral hepatitis is considered to be decreasing in Japan. On the other hand, recent lifestyle changes have led to an increase in the number of patients with non-viral chronic liver diseases, including alcoholic-related liver disease (ALD) and nonalcoholic fatty liver disease [9, 10]. Although recent advances in antiviral strategies and changing lifestyles are suggested to affect the etiology of LC in Japan, the classification system for Japanese public data [11] was determined based on the International Classification of Diseases (ICD)-10 [12], with some modifications, and the transition of the detailed etiology of LC is unclear.

In the annual meetings of the Japanese Society of Hepatology (JSH), a nationwide survey regarding the etiology of LC has been sporadically conducted; however, the last original report was related to an investigation performed in 2008 [13], although an original paper regarding a survey specifically for the etiology of non-viral LC was published in 2013 [14]. To assess the recent real-world changes in the etiology of LC in Japan, we conducted a nationwide survey in 2018 and compared the results to those from the 2008 survey.

## Patients and methods

### The diagnosis of liver cirrhosis and classification of etiologies

Since this survey was conducted as a special program in the JSH meeting, the diagnosis and etiological classification of LC were defined in accordance with the “Clinical Practice Guidelines for Management of Chronic Hepatitis and Cirrhosis 2016” [15] and “Textbook of Hepatology, 2nd Edition, 2016” [16], which were both edited by the JSH. Regarding the diagnosis of LC, the histological demonstration of pseudo-lobules in liver tissues was defined as the gold standard method. In addition, as histological examinations are invasive and expensive, a clinical diagnosis with the comprehensive evaluation of various clinical findings, including physical findings, laboratory tests, and imaging findings, was also acceptable for the diagnosis of LC in the clinical practice. The classification was as follows: (1) Hepatitis viral infection (HBV infection, HCV infection, and HBV + HCV co-infection); (2) ALD; (3) autoimmune hepatitis (AIH); (4) cholestasis; (5) hereditary metabolic diseases; (6) congestive liver diseases; (7) drug-induced liver injury; (8) specific infectious diseases; (9) nonalcoholic steatohepatitis (NASH); and (10) cryptogenic liver cirrhosis. Board-certified hepatologists of the JSH are referred to the above-mentioned books, determined the final diagnosis and classified the etiology of LC patients. Hepatitis viral infection was diagnosed according to the generally accepted serological criteria, including positivity for HBs antigen for HBV infection and positivity for HCV antibodies and HCV RNA. Regarding non-viral LC, the current classification requires attention to the following points: (1) LC due to primary biliary cholangitis (PBC) and primary sclerosing cholangitis has been classified as cholestasis-related LC; (2) Patients who were not able to be classified into either ‘ALD-related LC (alcoholic intake ≥ 60 g/day)’ or ‘NASH-related LC (alcoholic intake < 30 g in men and < 20 g in women)’ were classified as having ‘cryptogenic LC’; and (3) patients with overlapping AIH and PBC were classified as having AIH-related LC.

### The accumulation of LC patient data

A nationwide survey regarding the etiology of LC was conducted as the poster symposium of the 54th Annual Meeting of the JSH, which was held in June 2018, and 68 presenters throughout Japan with a nationwide geographical distribution showed data from a total of 79 hospitals. The cooperating institutions are listed in the “Appendix”. The current study aimed to assess the nationwide status of liver diseases, focusing on the changing distribution of etiologies of LC and was conducted in accordance with the Declaration of Helsinki 2013. The retrospective use of clinical records was approved by the ethics committees of the appropriate institutional review boards. Data were provided after each institution rechecked its own records, and the final results were confirmed in March 2019.

**Fig. 1:**
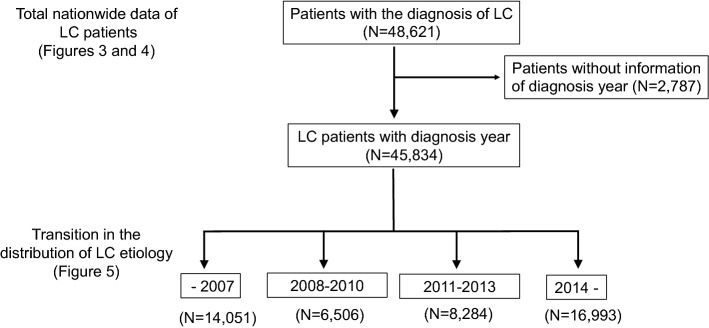
Flowchart of the current study. We first assessed the total nationwide data of all LC patients (*N* = 48,621). Then, we excluded the data of patients whose records did not include information on the year of diagnosis and evaluated the transition in the distribution of the etiologies of LC (*N* = 45,834). To assess the general trends in the etiologies of LC, we classified the patients into four groups (–2007, 2008–2010, 2011–2013, and 2014–)

A flowchart detailing the assessment of the distribution of etiologies is shown in Fig. 1. In accordance with the previous survey, we assessed the etiologies of all LC patients accumulated from all institutions (*N* = 48,621). We next analyzed the changing trends in the etiologies of LC in Japan by assessing cases for which the year of diagnosis was available (*N* = 45,834). The last published paper regarding the nationwide survey of all LC patients was based on the 2008 survey. Thus, we classified the data of patients diagnosed before 2008 (up to 2007) as one category. Then, the data of patients who were diagnosed in and after 2008 were further classified into three groups (diagnosed in 2008–2010, 2011–2013, and 2014–) according to the years of national surveys conducted by the MHLW (2008, 2011 and 2014) (Fig. 1).

**Fig. 2:**
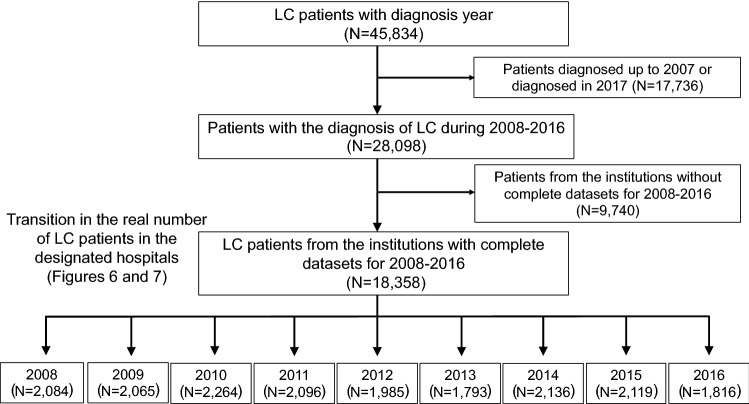
In the current survey, we attempted to assess the transition in the real number of patients newly diagnosed with LC. Most of the participating hospitals presented data up to 2016, as the complete data of 2017 were unavailable before the deadline for abstract submission (December 2017). Among the participating hospitals, 36 were able to provide the number of LC patients newly diagnosed each year from 2008 to 2016 without missing any years (*N* = 18,358). We analyzed the data to assess the transition of newly identified LC patients in the same hospitals in 2008–2016

In the current survey, we attempted to assess the transition in the real numbers of patients who were newly identified as having LC (Fig. 2). Most hospitals presented data up to 2016, as the complete data of 2017 were unavailable before the deadline for abstract submission (December 2017). Thus, we evaluated the data for 2008–2016. Among the participating hospitals, 36 were able to provide the number of LC patients newly diagnosed each year from 2008 to 2016 without missing any years. We analyzed the data of these 36 hospitals to assess the transition in the number of newly diagnosed LC patients in designated hospitals from 2008 to 2016.

### Statistical analyses

To compare the frequency of etiologies among multiple groups, data were analyzed by the chi-squared test. The groups with significantly higher or lower rates among all groups were subsequently determined by a residual analysis.

## Results

### Overall results for the etiologies of LC and geographic differences

**Fig. 3:**
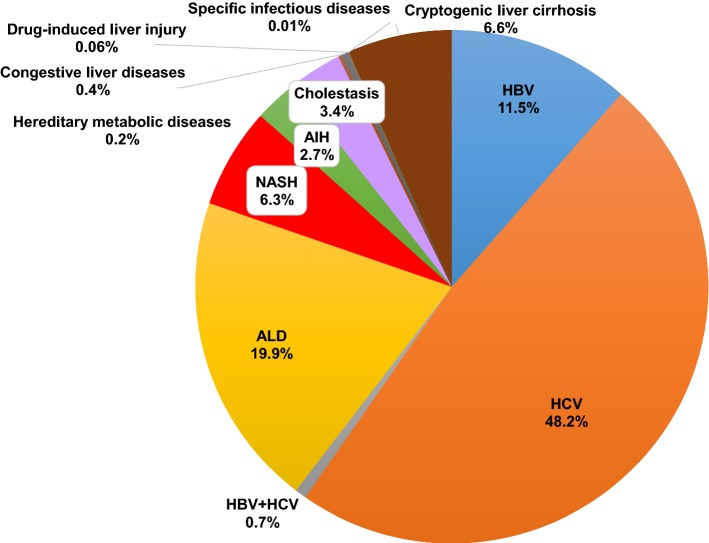
Overall results of the collected data on the etiologies of LC. A total of 48,621 cases were finally provided to analyze the etiologies of LC in Japan. In this cohort, 29,951 (61.6%) patients were men, and 18,670 (38.4%) were women. HCV infection was the leading cause of LC in Japan, and HBV infection (11.5%) was the third-most common cause. Although alcoholic-related liver disease (19.9%) and NASH (6.3%) were contributory causes of LC in Japan, viral hepatitis, particularly HCV, was the major cause

**Fig. 4:**
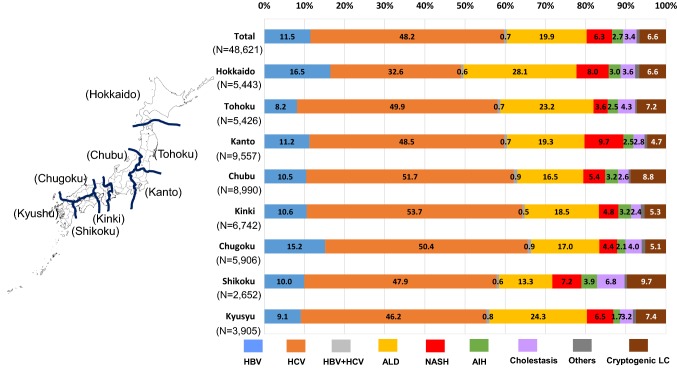
Geographic differences in the etiologies of LC. The etiologies of LC in all LC patients in the different geographic areas are shown. HCV-related LC was the leading cause of LC in all areas

A total of 48,621 cases were finally provided to analyze the etiologies of LC in Japan (Fig. 3). In this cohort, 29,951 (61.6%) patients were men, and 18,670 (38.4%) were women. HCV infection (48.2%) was the leading cause of LC in Japan, and HBV infection (11.5%) was the third-most common cause. Although ALD (19.9%) and NASH (6.3%) were contributory causes of LC in Japan, viral hepatitis, particularly HCV, was the major cause. The etiologies of the LC patients in different geographic areas are shown in Fig. 4. In the current study, we divided Japan into eight areas (Hokkaido, Tohoku, Kanto, Chubu, Kinki, Chugoku, Shikoku and Kyushu) according to the classification generally used in Japan (Fig. 4, left panel). HCV-related LC remained the leading cause of LC in all areas. The major results were as follows (Fig. 4, right panel); the ratios of HBV-related LC were high in the ‘Hokkaido’ (*p* < 1.0 × 10^−15^) and ‘Chugoku’ areas (*p* < 1.0 × 10^−15^), and low in the ‘Tohoku’ (*p* < 1.0 × 10^−15^) and ‘Kyushu’ areas (*p* < 1.0 × 10^−5^). The ratios of HCV-related LC were low in the ‘Hokkaido’ (*p* < 1.0 × 10^−15^) area. The ratios of ALD-related LC were high in the ‘Hokkaido’ (*p* < 1.0 × 10^−15^), ‘Tohoku’ (*p* < 1.0 × 10^−9^) and ‘Kyushu’ (*p* < 1.0 × 10^−12^) areas. The ratios of NASH-related LC were high in the ‘Hokkaido’ (*p* < 1.0 × 10^−7^) and ‘Kanto’ (*p* < 1.0 × 10^−15^) areas.

### The decreased involvement of viral hepatitis-related LC

We further assessed the transition in the etiologies of 45,834 LC cases (96.4%) for which the year of diagnosis was available (see Fig. 1). In the data from before 2008 (*N* = 14,051), the mean age was 63.8 years and 8708 (62.0%) patients were men. In the 2008–2010 data (*N* = 6506), the mean age was 66.4 years and 4082 (62.7%) patients were men. In the 2011–2013 data (*N* = 8284), the mean age was 67.4 years and 5153 (62.2%) patients were men. In the data from 2014 (*N* = 16,993) and thereafter, the mean age was 68.1 years and 10,309 (60.7%) patients were men (Table 1). There was no specific trend regarding gender ratio throughout the study period (Supplementary Fig. 1). In contrast, the mean age of the LC patients in Japan appeared to increase over time in the last decade (Supplementary Fig. 2).

**Table 1:** The data of the sex and mean age of the cirrhotic patients with diagnosis year (*N* = 45,834)

Diagnosis year (number of patients)	Total cases (*N* = 45,834)	–2007 (*N* = 14,051)	2008–2010 (*N* = 6506)	2011–2013 (*N* = 8284)	2014– (*N* = 16,993)
Men/women (%of men)	28,252/17,582 (61.6%)	8708/5343 (62.0%)	4082/2424 (62.7%)	5153/3131 (62.2%)	10,309/6684 (60.7%)
Mean age (years)	66.4	63.8	66.4	67.4	68.1

**Fig. 5:**
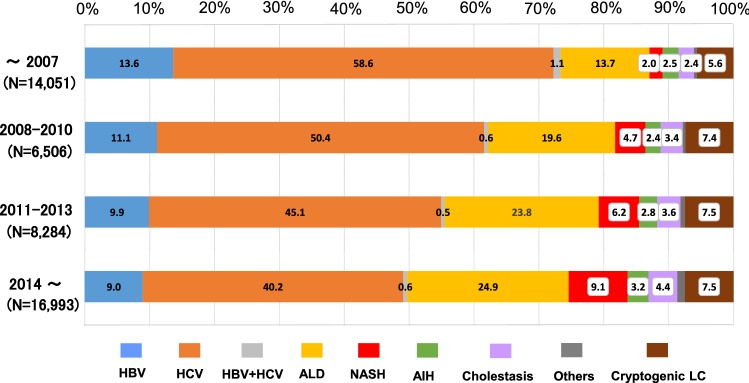
Transition of the distribution regarding the etiologies of LC. The transition in the distribution of the etiologies of LC. The ratio of HCV-related LC was notably decreased during the last decade, and that of HBV-related liver cirrhosis has also gradually declined. The ratio of non-viral LC surpassed that of HCV-related LC in patients who were diagnosed in and after 2014

The ratio of viral hepatitis-related LC was > 70% in the data from before 2008 (total, 73.4%; HBV alone, 13.6%; HCV alone 58.6%; and co-infection, 1.1%). In the 2008–2010 data, the ratio of viral hepatitis-related LC was 62.2% (HBV alone, 11.1%; HCV alone, 50.4%; and co-infection, 0.5%). The ratio tended to decrease over time and dropped below 50% in the data from 2014 and thereafter (total, 49.7%; HBV alone, 9.0%; HCV alone, 40.2%; and co-infection, 0.6%) (Fig. 5). Regarding LC of non-viral etiology, the ratios of ALD, NASH, AIH and cholestasis tended to increase (Fig. 5). In particular, ALD and NASH-related LC markedly increased from 13.7 to 24.9% and 2.0 to 9.1%, respectively.

**Fig. 6:**
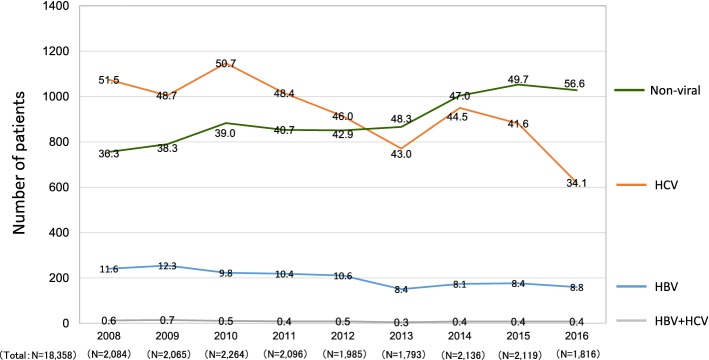
The transition in the number of patients who were diagnosed with LC. The numbers of newly diagnosed LC patients were identified according to the year of diagnosis, and a total of 18,358 patients were analyzed. The real numbers of patients with HBV-related and HCV-related LC decreased and the real number of patients with non-viral LC increased in 2008–2016. The percentages of the etiologies are shown in the graph bars

Although a decreased percentage of viral hepatitis-related LC was shown in Fig. 5, the results did not indicate a reduction in the number of viral hepatitis-related LC patients. To assess the transition of LC patients with the real numbers of patients, we analyzed the data of the 36 hospitals that were able to provide the number of LC patients newly diagnosed each year from 2008 to 2016 without missing any years (see Fig. 2). A total of 18,358 patients were included in this cohort (male, *N* = 11,441 [62.3%]; female, *N* = 6917 [37.7%]). In the 36 hospitals, the numbers of patients who were newly diagnosed with LC due to HBV-infection alone decreased from 241 (in 2008) to 160 (in 2016) (Fig. 6; blue line). The numbers of patients who were newly diagnosed with LC due to HCV-infection alone remarkably decreased from 1074 (in 2008) to 620 (in 2016) (Fig. 6; orange line). In contrast, the numbers of patients who were newly diagnosed with non-viral LC increased from 756 (in 2008) to 1028 (in 2016) (Fig. 6; green line). The ratio of viral hepatitis-related LC was > 60% in the 2008 data (total, 63.7%; HBV alone, 11.6%; HCV alone, 51.5%; and co-infection, 0.6%). The ratio tended to decrease and dropped below 50% in 2016 (total, 43.4%; HBV alone, 8.8%; HCV alone, 34.1%; and co-infection, 0.4%). The ratio of non-viral LC increased and surpassed that of HCV-related LC in 2013. In brief, the real number of patients newly diagnosed with viral hepatitis-related LC decreased, while the real number of patients newly diagnosed with non-viral LC increased from 2008 to 2016 in Japan.

### The transition of etiologies in non-viral LC patients

**Fig. 7:**
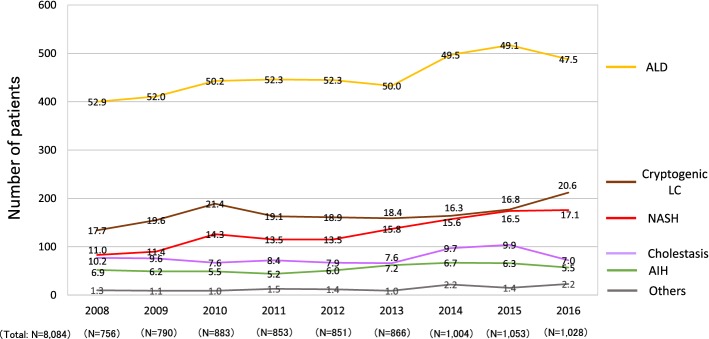
The transition in the number of patients who were diagnosed with non-viral LC. In agreement with the increase in the number of non-viral LC patients (Fig. 5), the complete sets of annual data from 36 hospitals revealed that the real numbers of patients newly diagnosed with alcoholic-related LC, NASH-related LC and cryptogenic LC were suggested to increase in 2008–2016. The percentages of the etiologies are shown in the graph bars

As shown in the Fig. 5, in accordance with the increase in the ratio of non-viral LC patients, the ratios of major non-viral LC etiologies in patients diagnosed in and after 2014 were higher than those in patients diagnosed before 2008, including ALD-related LC (24.9% vs. 13.7%), AIH-related LC (3.2% vs. 2.5%), cholestasis-related LC (4.4% vs. 2.4%), NASH-related LC (9.1% vs. 2.0%) and cryptogenic LC (7.5% vs. 5.6%). We analyzed the data of the non-viral LC patients of the Fig. 6 in more detail and assessed the transition in the real number of new patients with non-viral LC (Fig. 7). The number of patients newly diagnosed with ALD-related LC showed a small increase from 400 (in 2008) to 488 (in 2016) (Fig. 7; gold line). The number of patients newly diagnosed with NASH-related LC showed a marked increase from 83 (in 2008) to 176 (in 2016) (Fig. 7; red line). The number of patients diagnosed with cryptogenic LC increased from 134 (in 2008) to 212 (in 2016) (Fig. 7; brown line). Thus, the real numbers of patients who were newly diagnosed with ALD-related LC, NASH-related LC and cryptogenic LC in Japan were suggested to have increased from 2008 to 2016.

## Discussion

The present report showed the changing etiology of LC in Japan. To assess the recent real-world changes in the etiologies of LC in Japan, we conducted a nationwide survey in 2018 and the results were compared with those of the 2008 survey. Although nationwide surveys have been conducted in JSH annual meetings, these surveys have been sporadic and have had their own diagnostic criteria [13]. This is the first study in which nationwide experts referred to the same criteria and determined the transition in the etiologies of LC in Japan. Our results suggested a recent change in the etiology of LC, with a decreased contribution of hepatitis virus-associated LC and an increased contribution of non-viral LC.

Regarding the etiologies of LC (Fig. 3), the total ratio of viral hepatitis-related LC was > 60%. When our results were compared to those in 2008, HBV-related LC and HCV-related LC decreased from 13.9 to 11.5% and 60.9 to 48.2%, respectively. The ratio of non-viral LC increased, the ratio of LC due to ALD increased from 13.6 to 19.9%, and that of NASH-related LC also increased from 2.1 to 6.3%. In addition, cholestasis-related LC increased slightly from 2.7% (PBC 2.4% and other cholestasis 0.3% in 2008) to 3.4%, and AIH also showed a small increase from 1.9 to 2.7%. These findings suggested a decreased ratio of viral hepatitis-related LC and an increased ratio of non-viral LC in Japan.

The data of the present study were generally consistent with the results of the 2008 survey. Regarding the LC patients diagnosed before 2008 in the current study, the ratios of HBV-related LC (13.7%), HCV-related LC (58.6%) and NASH-related LC (2.0%) were similar to those of the previous survey (HBV-related LC, 13.9%*;* HCV-related LC, 60.9%; NASH-related LC, 2.1%) [13]; however, the ratio of cryptogenic LC patients diagnosed before 2008 (5.6%) was higher relative to the 2008 survey (3.0%) [13]. One possible explanation is that differences in the participating institutions influenced the results. Additionally, we have to note that each survey had its own classifications for LC patients, particularly for non-viral LC patients. The survey in 2008 classified non-viral LC into 10 categories, while the current study classified non-viral LC into nine etiologies. Thus, methodological differences between the two surveys might have somehow been involved in the discrepancy.

Regarding the geographic differences in the etiologies of LC patients, HCV-related LC remained the leading cause of LC in all areas (Fig. 4), and the characteristics of the etiologies in each area were generally in agreement with the previous survey. However, some differences were observed between the 2008 and 2018 surveys. In our results, the ratio of HBV-related LC seemed to be high in the ‘Kanto’ area. Additionally, the ratio of ALD-related LC was high in the ‘Kyushu’ area. We should again mention the methodological differences between the surveys. In the previous survey, the ‘Chubu’ area was divided into the ‘Hokuriku’ and ‘Chubu’ areas. In addition, ‘Tokyo’ had been categorized as one independent area separately from the ‘Kanto’ area, while ‘Okinawa’ prefecture was considered separately from the ‘Kyushu’ area. Similarly to the above-mentioned discrepancy regarding the etiologies of non-viral LC, some methodological differences may have caused the different results.

Overall, the comparison of the 2008 and 2018 surveys suggested a decreased ratio of viral hepatitis-related LC, particularly HCV-related LC and an increased ratio of non-viral LC, including NASH-related LC. Our results were consistent with those of previous reports that have suggested that non-viral liver diseases are increasingly contributing as etiologies of chronic liver diseases, such as the changing etiologies of hepatocellular carcinoma in Japan as well the United States of America [17–19]. To represent the recent real-world data, all data used in this study were obtained from geographical regions or areas throughout Japan. Even with the presence of some slightly different data due to methodological differences, we believe that the comparison of the results among multiple surveys could be beneficial for assessing the transition in the etiologies of LC. However, we also consider that caution is required when interpreting the results of comparisons among multiple surveys.

One characteristic point of the current survey was that nationwide experts referred to the same criteria and the transition in the etiology of LC patients were evaluated according to the defined standards. In addition, we assessed the change not only in the distribution of etiologies (Fig. 5) but also in the real numbers of new LC patients (Figs. 6 and 7). Regarding the data on the proportions of etiologies (Fig. 5), the proportions of non-viral LC, including ALD-related and NASH-related LC, seemed to have increased in the last decade. However, the results did not demonstrate an increase in the number of non-viral LC patients in Japan. According to the survey on the real numbers of patients (Fig. 6), the number of patients who were newly diagnosed with non-viral LC increased, while the number of patients newly diagnosed with viral hepatitis-related LC, especially HCV-related LC, decreased over time, suggesting that both the decrease in the viral hepatitis-related LC and the increase in non-viral LC contributed to changing the distribution of the LC etiologies in Japan (Fig. 5). Although some papers that evaluated the real numbers of LC patients reported survey results in specific regions of Japan or China [20, 21], the current cohort is the largest yet and describes the real-world nationwide data in Japan.

As described in “Introduction”, Japan has a high prevalence of viral hepatitis, and even before the establishment of the GHSS on viral hepatitis, various measures for the prevention and treatment of viral hepatitis were intensively implemented under national programs [5]. We consider that various national strategies implemented since 2008 as well as advances in antiviral treatments have helped to reduce the rate of hepatitis virus-related disease, since HCV elimination due to antiviral treatments, such as interferon or direct-acting antivirals (DAAs), could prevent progression to LC in HCV-infected patients. HCV infection was widespread around 60 years ago in Japan; thus, Japanese patients have been suffering from this disease for longer in comparison to patients in other countries, suggesting that the transition of Japanese patients with HCV-related liver diseases could help to identify future global trends in HCV-related diseases [22]. In addition, HCV-infected patients tend to be elderly in Japan, and the number of patients is expected to decrease due to the improved antiviral treatment in combination with a natural reduction over time [23]. Our real-world data demonstrating a reduced contribution of HCV infection to the etiology of LC in Japan may help predict the future trends in other countries.

The present study was associated with several limitations. First, this survey was conducted according to two JSH-edited books. Although HBV and/or HCV infection could be serologically diagnosed, the diagnostic criteria for other non-viral liver diseases, such as NASH, were not completely unified. In addition, the guideline was published for a wide range of physicians. Thus, the classification was determined according to the clinical diagnosis, and histological assessment was not mandatory in the current survey. Although our results were generated under the standards defined by hepatologists, care should be taken when applying our results to other countries, especially for data regarding the etiology of non-viral LC. Second, the current survey was focused on the changing trends in the etiology of LC and we did not request detailed clinical data. This simplicity was advantageous for accumulating a large number of LC patients; however, we could not scrupulously report the clinical features of LC patients. Regarding the age of LC patients, since the data available in the current survey were limited to the mean age of LC patients from each institute, we could only show the increasing trend in the mean age (Supplementary Fig. 2), even though analyzing the background characteristics, such as sex and mean age of the LC patients, according to the etiology would have been more useful. Third, the retrospective data were obtained directly from the participating institutions, and older data were difficult to retrieve in a number of institutions. Our clinical data with the real number of patients (Figs. 6 and 7) were obtained from 36 hospitals without any missing or incomplete data. Although these hospitals were located in all areas of Japan, it should be noted that the results were obtained from a limited number of enrolled patients (*N* = 18,358; Fig. 2). In addition, the present study employed a retrospective design. The transition of patient numbers according to the etiology should be prospectively examined among the same institutions. We analyzed the data of the 36 hospitals that were able to provide the number of LC patients newly diagnosed each year from 2008 to 2016 without missing any years because these hospitals were considered to have continuously accumulated their own data independently of the current survey, suggesting that their data were simulated the prospective cohort to some extent. However, our results were not equivalent to prospective data.

In summary, we showed the survey results regarding the transition in the etiologies of LC in Japan. Our results suggested a decreased ratio of hepatitis virus-associated LC, particularly HCV-related LC and an increase in the ratio of non-viral LC, including NASH-related LC in the real world.

### Electronic supplementary material

Below is the link to the electronic supplementary material.

Supplementary file1 (DOCX 209 kb)

## Appendix

We again appreciate the following institutions that participated in the poster symposium of the 54th Annual Meeting of the JSH.

Akita University Graduate School of Medicine; Anjo Kosei Hospital; Asahikawa City Hospital; Asahikawa Medical University; Chiba University, Graduate school of medicine (Gastroenterology); Dokkyo Medical University Saitama Medical Center; Ehime University Graduate School of Medicine (Department of Gastroenterology and Metabology); Fukushima Medical University School of Medicine (Department of Gastroenterology); Gunma University Hospital; Hakodate Municipal Hospital; Hiroshima Red Cross and Atomic Bomb Survivors Hospital; Hiroshima University Hospital (Gastroenterology and Metabolism); Hokkaido P.W.F.A.C. Asahikawa-Kosei General Hospital; Hokkaido University Hospital (Department of Gastroenterology and Hepatology); Hyogo College of Medicine (Division of Hepatobiliary and Pancreatic Diseases, Department of Internal Medicine); Hyogo Prefectural Nishinomiya Hospital; Ikeda Municipal Hospital (Department of Gastroenterology); Isesaki Municipal Hospital; Iwate Medical University; Japanese Red Cross Asahikawa Hospital; Japanese Red Cross Date Hospital (Department of Gastroenterology); Jichi Medical University (Department of Medicine, Division of Gastroenterology and Hepatology); Juntendo University Shizuoka Hospital (Gastroenterology and Hepatology); Kagawa Prefectural Central Hospital (Department of Hepatology); Kagawa University (Department of Gastroenterology and Neurology); Kagoshima University Graduate School of Medical and Dental Sciences (Digestive and Lifestyle Diseases, Department of Human and Environmental Sciences); Kanazawa University (Department of Internal medicine/Hepatology and Gastroenterology); Kansai Rosai Hospital; Kawasaki Municipal Tama Hospital; Keio University Hospital; Kiryu Kosei General Hospital; Kumamoto University, Graduate School of Medical Sciences (Department of Gastroenterology and Hepatology); Kurume University School of Medicine (Division of Gastroenterology, Department of Medicine); Kushiro Rosai Hospital; Kusunoki-Hospital; Kyoto Prefectural University of Medicine Graduate School; Maebashi Red Cross Hospital; Mie University Graduate School of Medicine; Mie-chuo Medical Center; Musashino Red Cross Hospital; Nagoya City University Graduate School of Medical Sciences (Department of Gastroenterology and Metabolism); Nara Medical University (Department of 3rd Internal Medicine); National Hospital Organization Kyoto Medical Center (Department of Gastroenterology); Nayoro City General Hospital; Nihon University school of Medicine (Division of Gastroenterology and Hepatology, Department of Internal Medicine); Niigata University, Graduate School of Medical and Dental Sciences (Division of Gastroenterology and Hepatology); Nippon Life Hospital; Ogaki Municipal Hospital; Oita University, Faculty of Medicine (Department of Gastroenterology); Okayama City Hospital (Department of Gastroenterology); Okayama University Graduate School of Medicine, Dentistry and Pharmaceutical Sciences (Department of Gastroenterology and Hepatology); Ome Municipal General Hospital; Osaka City University, Graduate School of Medicine (Department of Hepatology, Department of Endowed Department of Liver Cirrhosis Therapeutics and Department of Premier Preventive Medicine); Osaka Police Hospital; Public Tomioka General Hospital; SAGA university (Division of Metabolism and Endocrinology Depertment of Internal Medicine); Saga-Ken Medical Centre Koseikan; Saiseikai Niigata Daini Hospital; Saitama Medical Center; Saitama Medical University; Sapporo Kosei General Hospital; Shibukawa Medical Center; Shiga University of Medical Science (Department of internal medicine, gastroenterology),; Shimane University Hospital; Shinshu University School of Medicine; Takasaki General Medical Center; Teine Keijinkai Hospital; Tohoku University Hospital; Tokyo Kyosai Hospital; Tokyo Medical University Ibaraki Medical Center; Tokyo Women's Medical University (Department of Internal Medicine and Gastroenterology); Tomakomai City Hospital; Toranomon Hospital; Tottori University Hospital; University of Fukui, School of Medical Sciences (Second Department of Internal Medicine); University of Ryukyus; University of Yamanashi, Faculty of Medicine (First Department of Internal Medicine); Yamagata University, Faculty of Medicine (Department of Gastroenterology); Yamaguchi University Graduate School of Medicine (Department of Gastroenterology and Hepatology).

## Abbreviations

LC: Liver cirrhosis
HBV: Hepatitis B virus
HCV: Hepatitis C virus
GHSS: Global Health Sector Strategy
WHO: World Health Organization
MHLW: Ministry of Health, Labour and Welfare
ALD: Alcoholic-related liver disease
ICD: International Classification of Diseases
JSH: Japanese Society of Hepatology
NASH: Nonalcoholic steatohepatitis
PBC: Primary biliary cholangitis
DAA: Directly acting antiviral
