# Supplementary material for: Transition in the etiology of liver cirrhosis in Japan: a nationwide survey
Source: J Gastroenterol. 2019 Nov 25;55(3):353–62. doi: 10.1007/s00535-019-01645-y (PMC7026312; doi:10.1007/s00535-019-01645-y)
Supplement: Supplementary file 1 — Supplementary file1 (DOCX 209 kb) [file 535_2019_1645_MOESM1_ESM.docx]

**Supplementary Material**

Enomoto H, et al.; Transition in the etiology of liver cirrhosis in Japan: A nationwide survey

**Supplementary Figure 1: Transition in the distributions of men and women among cirrhotic patients**

**
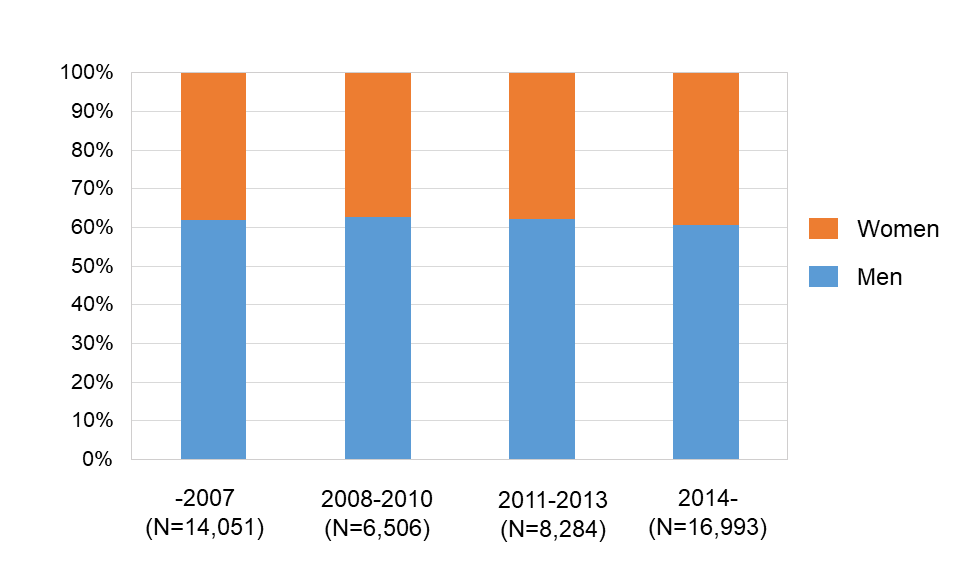
**

The distributions of men and women (**Table 1**) are graphically shown. There was no specific trend throughout the period. The diagnosis years and numbers of patients (N) are shown below the graph.

**Supplementary Figure 2: Trends in the mean age of cirrhotic patients using the moving average analysis**


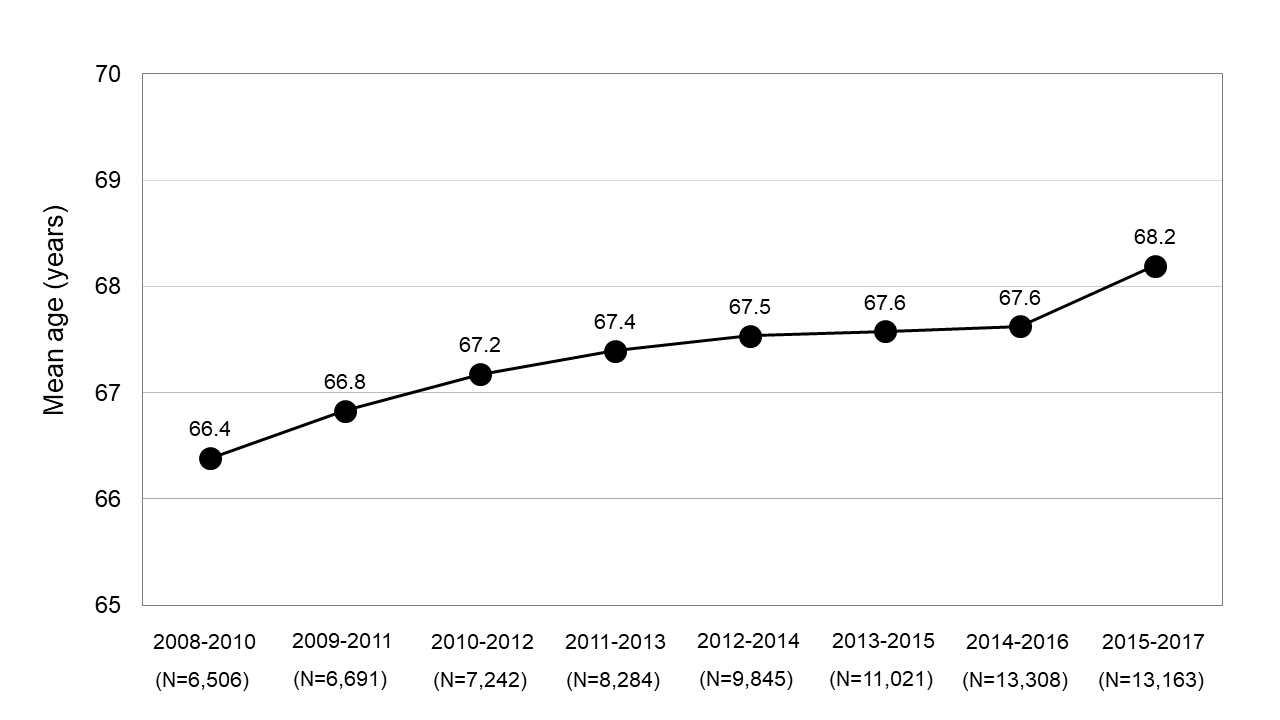


The three-year moving averages were calculated and shown. The mean age of the cirrhotic patients was suggested to have increased during the last decade. The diagnosis years and numbers of patients (N) are shown below the graph.
